# Altered Cardiovascular Reactivity to and Recovery from Cold Face Test-Induced Parasympathetic Stimulation in Essential Hypertension

**DOI:** 10.3390/jcm10122714

**Published:** 2021-06-19

**Authors:** Lisa-Marie Walther, Roland von Känel, Nadja Heimgartner, Claudia Zuccarella-Hackl, Ulrike Ehlert, Petra H. Wirtz

**Affiliations:** 1 Biological Work and Health Psychology, University of Konstanz, 78457 Konstanz, Germany; lisa-marie.walther@uni-konstanz.de; 2 Centre for the Advanced Study of Collective Behaviour, University of Konstanz, 78457 Konstanz, Germany; 3 Department of Consultation-Liaison Psychiatry and Psychosomatic Medicine, University Hospital Zurich, University of Zurich, 8091 Zurich, Switzerland; roland.vonkaenel@usz.ch (R.v.K.); claudia.hackl-zuccarella@usz.ch (C.Z.-H.); 4 Division of Clinical Psychology and Psychotherapy, University of Basel, 4055 Basel, Switzerland; nadja.heimgartner@unibas.ch; 5 Department of Clinical Psychology and Psychotherapy, University of Zurich, 8050 Zurich, Switzerland; u.ehlert@psychologie.uzh.ch

**Keywords:** hypertension, parasympathetic stimulation, Cold Face Test (CFT), cardiovascular reactivity, chronic stress

## Abstract

Essential hypertension is associated with increased sympathetic and diminished parasympathetic activity as well as impaired reactivity to sympathetic stimulation. However, reactivity and recovery from parasympathetic stimulation in hypertension are unknown. We investigated reactivity and recovery to primarily parasympathetic stimulation by Cold Face Test (CFT) in essential hypertension. Moreover, we tested whether chronic stress modulates CFT-reactivity dependent on hypertension status. The CFT was conducted by applying a cold face-mask for 2 min in 24 unmedicated, otherwise healthy hypertensive men and in 24 normotensive controls. Systolic and diastolic blood pressure (BP) and heart rate (HR) were measured repeatedly. Chronic stress was assessed with the Trier-Inventory-for-Chronic-Stress-Screening-Scale. Hypertensives did not exhibit diastolic BP decreases after CFT-cessation (*p* = 0.59) as did normotensives (*p* = 0.002) and failed to show HR decreases in immediate response to CFT (*p* = 0.62) when compared to normotensives (*p* < 0.001). Systolic BP reactivity and recovery patterns did not differ between hypertensives and normotensives (*p* = 0.44). Chronic stress moderated HR (*p* = 0.045) but not BP CFT-reactivity (*p*′s > 0.64) with chronically stressed normotensives showing similar HR reactivity as hypertensives. Our findings indicate impaired diastolic BP and HR reactivity to and recovery from CFT in hypertensives and a moderating effect of chronic stress on HR reactivity potentially reflecting reduced relaxation ability of the cardiovascular system.

## 1. Introduction

Arterial hypertension, characterized by chronically elevated arterial blood pressure (BP), is a major risk factor for coronary heart disease [1]. About 95% of hypertensive patients are diagnosed as “essential hypertensives” as the cause for their condition is unknown [2]. Essential hypertension and its pathogenesis have been associated with autonomic dysfunction manifested by increased sympathetic nervous system (SNS) activity and reactivity to stimulation as well as concurrent diminished parasympathetic nervous system (PNS) activity [3].

So far, activity and especially reactivity of the PNS in essential hypertension has not been well studied. Evidence suggesting reduced basal parasympathetic activity in hypertension comes from few studies assessing heart rate variability (HRV) [4,5] and salivary flow [6] under resting conditions. Reactivity to *parasympathetic inhibition* in hypertension was first investigated by Julius et al. [7]. In their study, borderline hypertensives (HT) previously treated with the SNS antagonist propranolol exhibited comparably smaller increases in heart rate (HR) and cardiac output after atropine-induced PNS blockade when compared to identically treated normotensives (NT), interpreted as diminished reactivity to parasympathetic inhibition. These results were confirmed for essential HT and also extended to BP reactivity [8]. Similarly, borderline HT had a less pronounced decrease in salivary flow after intravenous injection of atropine compared to NT [9] and HT were found to display attenuated HR recovery after exercise [10]. With respect to *parasympathetic stimulation*, reactivity in HT in terms of either salivary flow rate, or HR, HRV and BP seems to be impaired as was evidenced by use of the PNS stimulant neostigmine [9] and slow/deep breathing [11,12,13].

A non-invasive and simple method to reliably provoke parasympathetic stimulation is the Cold Face Test (CFT) [14,15]. The CFT comprises application of a cooled mask to the face. This cold stimulus induces the characteristic autonomic changes that occur with the diving reflex, i.e., primarily PNS stimulation in terms of trigeminal–vagal mediated bradycardia and minor SNS co-stimulation by trigeminal–sympathetic mediated peripheral vasoconstriction, but without impairment of breathing [14,15,16,17]. Notably, the CFT differs from the Cold Pressure Test [18] often applied in the context of hypertension, where hand or foot immersion in ice water for 1 min leads to a pressor response and thus stimulation of the SNS [19]. Additionally, the SNS activation component of CFT exposure is assumed to be lower as compared to that of the diving reflex since the latter has been associated with the apnea when diving [20]. In immediate response to 1 min CFT-application, i.e., 30 to 60 s after onset, borderline HT displayed significant increases in systolic BP (SBP) but not diastolic BP (DBP) or HR, and lower HRV, while NT showed HR decreases without concomitant changes in BP [21]. However, following 5 min CFT-conduction HT did show bradycardia, although to a lower extent than NT, whereas no group difference was observed for HRV response [22]. Cardiovascular recovery from CFT stimulation has not previously been investigated, neither in NT nor in hypertension. 

Chronic stress is hypothesized to play a role in the development of hypertension [23]. According to the allostatic load concept, repeated stressful challenges can result in chronic activation of stress-responsive physiological systems which may accumulate over time and result in chronically elevated BP [24,25]. Indeed, empirical evidence links chronic stress with the sustained elevation of BP [26]. In reaction to sympathetic stimulation, chronic stress relates to altered cardiovascular reactivity and impaired recovery [27]. Moreover, chronic stress was associated with diminished basal parasympathetic activity [28] comparably to HT [4,5]. In the context of PNS stimulation, chronic stress has not yet been investigated. 

The aim of the present study was to investigate cardiovascular reactivity to and recovery from stimulation by CFT [14,16] in unmedicated, otherwise healthy, hypertensive men compared to normotensive controls. We repeatedly measured SBP, DBP, and HR before and up to 10 min after CFT. Based on the above-summarized literature, we hypothesized group differences in terms of attenuated BP and HR reactivity and consequently flatter recovery in HT as compared to normotensive controls. Moreover, we investigated whether chronic stress modulates cardiovascular reactivity to CFT. We specifically expected that NT with higher chronic stress resemble the cardiovascular CFT-reactivity of HT. 

## 2. Materials and Methods

### 2.1. Study Participants

With the aid of the Swiss Red Cross of the Canton of Bern and the Clinical Investigation Unit of the University Hospital of Bern/Inselspital, we recruited hypertensive and normotensive men between 20 and 65 years who, apart from having hypertension, were healthy and medication-free. Specifically, members of our study team accompanied the Swiss Red Cross mobile blood donation unit that routinely assesses BP before blood donation. Male blood donors with elevated BP (SBP ≥ 140 mmHg and/or DBP ≥ 90 mmHg) expressing interest in study participation were asked to provide an initial BP diagnostic by home assessment as part of the assessment of essential hypertension (see below). For each hypertensive participant, we recruited a normotensive control of similar age on a case-by-case basis. Participation was restricted to male subjects, in particular because of gender differences in vagal activity and autonomic control of the heart [29]. Additional specific exclusion criteria, verified in a structural clinical screening on the study day, included: any regular or current prescribed or non-prescribed medication intake, psychopathology or psychiatric diseases, respectively, alcohol abuse and illicit drug use, smoking, any heart disease, varicosis and thrombotic diseases, elevated blood sugar levels and diabetes, elevated cholesterol levels, liver and renal diseases, chronic obstructive pulmonary disease, allergies and atopic diathesis, rheumatic diseases, cancer, chronic pain, sleep disturbances, thyroid disease, current infectious diseases, and secondary hypertension. 

The study was carried out in accordance with the Declaration of Helsinki principles and formally approved by the Ethics Committee of the Canton of Bern, Switzerland (154/07; 07.09.09) and the Swiss Agency for Therapeutic Products (Swissmedic). All participants provided written consent before participating. 

### 2.2. Assessment of Essential Hypertension

For the assessment of hypertension, we applied a two-step assessment procedure. 

#### 2.2.1. Home BP Assessment

Participants provided an initial BP diagnostic by home assessment using sphygmomanometry (Omron IntelliSense M6, Omron Healthcare Europe B.V., Hoofdorp, The Netherlands). Following written instructions, each participant was required to measure BP in seated position after a minimum of 15 min rest twice per day (once in the morning and once in the evening) on up to 3 separate days. We computed the average home BP as initial BP diagnostic with participants conservatively categorized as preliminarily hypertensive following the European Society of Hypertension (ESH) recommendations for home BP measurements (hypertension: home assessed SBP ≥ 135 mmHg and/or DBP ≥ 85 mmHg) [30]. Participants were considered preliminarily normotensive if their home assessed SBP was below 135 mmHg and DBP below 85 mmHg. 

#### 2.2.2. Study BP Assessment

The preliminary categorization was extended by the mean of two additional seated study BP measurements performed using automated sphygmomanometry (Hewlett-Packard 78352C, Hewlett-Packard GmbH, Böblingen, Germany) during the clinical screening on the study day, each after 15 min rest. The categorization of hypertension according to study BP measurements was carried out following the World Health Organization (WHO)/International Society of Hypertension definition (hypertension: SBP ≥ 140 mmHg and/or DBP ≥ 90 mmHg) [31]. Notably, we considered participants as normotensive according to study BP measurements if their SBP was below 140 mmHg and DBP below 90 mmHg. 

We a priori calculated a sample size of 48 participants (see below). Of a total of 55 recruited persons, 7 had to be excluded; 5 failed to meet a clear categorization of hypertension/normotension, e.g., due to inconsistent home vs. study BP categorization and 2 did not complete the study due to BP and HR measurement failure during CFT (see below). Of the final study sample of 24 HT and 24 NT, 6 HT and 5 NT did not provide home BP measurements. To maintain the two-step BP assessment procedure, we therefore substituted the missing home BP measurements by the baseline BP measurement before CFT (see hemodynamic measures) to verify study-measurement based categorization. 

Assessment of serum creatinine, calcium, sodium, potassium, HbA1c, and low-density lipoprotein/high-density lipoprotein ratio on the study day would have allowed post hoc exclusion of participants with secondary hypertension and therefore diagnosis of essential hypertension in all eligible hypertensive participants. However, no participant had to be excluded due to secondary hypertension. Moreover, our two-step assessment procedure also allowed to exclude white coat hypertension in our participants. 

We calculated resting BP as the mean of the two seated study BP measurements to obtain a continuous measure for hypertension. 

### 2.3. Procedure 

Participants were asked to abstain from excessive sports activities and consumption of caffeinated and alcoholic beverages 24 h prior to study participation. The study was performed in the Clinical Investigation Unit of the Bern University Hospital (Inselspital). After their arrival between 9:00 AM and 4:30 PM, participants first completed a clinical screening to assess study eligibility followed by a physical examination assessing participants’ height and weight. Further, they provided a blood sample after a resting period of at least 15 min to retrospectively verify essential hypertension and exclude potential secondary hypertension. Afterwards, the study procedure started. After a 25 min resting period, the CFT was conducted. Prior to dismission, participants completed various psychological questionnaires.

### 2.4. Cold Face Test (CFT)

To provoke parasympathetic stimulation, we conducted the CFT which mimics the diving reflex in particular by inducing primarily trigeminal–vagal-mediated bradycardia accompanied by trigeminal–sympathetic-mediated peripheral vasoconstriction [14,15,16]. A full-face mask (Dr. Winkler GmbH, Ainring-Mitterfelden, Germany) with openings for eyes avoiding an oculocardiac reflex and openings for nose and mouth allowing normal breathing, was placed on the face of the sitting participants for 2 min. The temperature of the cold mask was 1 °C. To ensure a steady temperature of 1 °C during the period of 2 min, an additional cold pack (Nexcare, 3M Health Care, St. Paul, MN, USA) was affixed to the mask. Subjects were instructed in advance to continue normal breathing and abstain from moving or talking during CFT. 

### 2.5. Hemodynamic Measures

BP and HR were assessed in seated position on the dominant arm using automated sphygmomanometry (Hewlett-Packard 78352C, Hewlett-Packard GmbH, Böblingen, Germany). Five measurements were made to investigate CFT reactivity comprising 1 baseline measurement 3 min before start of CFT, 1 measurement 1.5 min after onset, i.e., during CFT (immediate CFT-reactivity), and 3 post-CFT measurements at 3, 5, and 10 min after CFT-cessation (recovery), respectively. 

### 2.6. Psychological Assessment

Psychological assessment was performed to (1) verify participants’ mental health, (2) to assess their levels of chronic stress, and (3) to investigate potential associations between chronic stress and reactivity to parasympathetic stimulation. 

#### 2.6.1. Psychopathology 

To assess mental health, we tested for general psychopathology using the Brief Symptom Inventory (BSI) [32]. The BSI contains 58 items about the frequency and severity of strain resulting from somatization (7 items), obsessive-compulsivity (6 items), interpersonal sensitivity (4 items), depression (6 items), anxiety (6 items), hostility (5 items), phobic anxiety (5 items), paranoid ideation (5 items), and psychoticism (5 items). In total, 4 additional items target loss of appetite, sleeping problems, and suicidal thoughts. Items are rated on a 5-point rating scale ranging from 0 (“not at all”) to 4 (“very much”). By evaluating the average of all item ratings, the Global Severity Index (GSI) representing the general current distress is obtained. Possible GSI scores range from 0 to 4 with higher scores indicating a higher level of current distress. Data of 1 HT participant were missing due to incompletion. For the GSI scale, Cronbach’s α was 0.90 in our sample. 

#### 2.6.2. Chronic Stress 

To assess participant’s chronic stress, we applied the 12-item Chronic Stress Screening Scale of the Trier Inventory for Chronic Stress (TICS-CSSS) [33]. The TICS-CSSS comprises questions about the frequency of experienced work overload (4 items), worries, (4 items), lack of social recognition (2 items), excessive demands at work (1 item), and social overload (1 item) within the last 3 months. Items are rated on a 5-point scale (0 = “never” to 4 = “very often”) with a total score ranging from 0 to 48. Higher scores indicate greater levels of chronic stress. Data of 1 HT and 3 NT participants were missing due to incompletion. Cronbach’s α for TICS-CSSS was 0.93 in our sample. 

### 2.7. Statistical Analyses

Data were analyzed using SPSS (Version 26.0) statistical software packages for Macintosh (IBM SPSS Statistics, Chicago Il, USA). All analyses were two-tailed with the level of significance set at *p* < 0.05. Results with significance levels *p* < 0.10 were considered as marginally significant. Missing data were list-wise excluded for the respective parameter. Results are presented as mean ± standard error of the mean (*M ± SEM*). Sigma Plot (Version 13; Systat Software GmbH, Erkrath, Germany) was used for graphics creation. We a priori calculated power analyses using the statistical software G*Power for Macintosh (Version 3.1.9.6; Heinrich Heine University Düsseldorf, Germany) [34]: the optimal sample size to detect interactions between group and repeated hemodynamic parameters given an expected small effect size of *f* = 0.10, an expected observed average correlation of the repeated measures of *r >* 0.85, α = 0.05, and a power of 0.90 is *n* = 48. 

Prior to statistical analysis, all data were tested for normal distribution and homogeneity of variance using Kolmogorov–Smirnov and Levene tests. As assumption of normality was not met for HR data, HR data were transformed using the natural logarithm and homogeneity of variance was verified. For reasons of clarity, original data are presented in the figures. In order to protect against violations of sphericity, we applied Huynh-Feld correction where appropriate. Body mass index (BMI) was calculated by the formula BMI = kg/m^2^. Mean resting arterial BP (MAP) was calculated by the formula MAP = (2/3×resting DBP) + (1/3×resting SBP). In HR data analyses, 6 participants had to be excluded due to problems with HR assessment, i.e., incomplete data or problems with baseline HR assessment. 

To test for group differences in demographic, resting, and baseline physiological as well as psychological measures, we used univariate analyses of variance (ANOVA). To test for group differences in CFT-induced reactivity, we calculated repeated measures ANOVAs with group (HT vs. NT) as the independent variable and repeated SBP, DBP, or HR levels as repeated dependent variables. Complementarily, we tested for linear associations between CFT-induced reactivity and MAP as a continuous measure of hypertension assessment by calculating the same repeated ANOVAs using MAP as a continuous independent variable instead of group. Post-hoc tests comprised univariate ANOVAs for each measurement time point while controlling for the respective baseline, repeated measures ANOVAs between baseline and every later measurement time point, and/or separate analyses of repeated measures ANOVAs in each group. Due to the potentially confounding effects of age on autonomic activity, we additionally performed all repeated AN(C)OVAs controlling for age as a covariate [35]. Moreover, as obesity is associated with ANS dysfunction in terms of increased SNS (re)activity and decreased PNS activity [36], we also controlled for BMI. 

To test for associations between chronic stress and hemodynamic CFT-reactivity, we calculated general linear models (GLM) with repeated SBP, DBP, or HR levels as dependent variables, group (HT vs. NT) as categorical independent variable, chronic stress as continuous independent variable, and the interaction term between group and chronic stress. Notably, group and chronic stress were Z-transformed prior to computation of interaction terms. Again, we performed all GLMs with and without controlling for age and BMI as potentially confounding covariates. 

To graphically illustrate our findings, we performed a median split on the TICS-CSSS rendering four subgroups, i.e., HT with lower chronic stress, HT with higher chronic stress, NT with lower chronic stress, and NT with higher chronic stress. 

Effect size parameters (*f*) were calculated from partial eta squared (η^2^) using G*Power for Macintosh (Version 3.1.9.6; Heinrich Heine University Düsseldorf, Germany) and are reported where appropriate (effect size conventions: *f* 0.10 = small, 0.25 = medium, 0.40 = large) [37]. 

## 3. Results

### 3.1. Participants’ Characteristics

Our final sample comprised a total of 48 participants, 24 hypertensive participants and 24 normotensive controls. As expected, HT displayed significantly higher average resting SBP, DBP, and MAP compared to NT. In addition, HT had a higher BMI than NT. The two groups did not significantly differ in terms of average resting HR, age, or any psychological measure (*p*′s ≥ 0.40). Participants’ characteristics are depicted in Table 1.

### 3.2. CFT-Induced Reactivity

As expected and in line with average resting measurements, HT had higher baseline SBP and DBP (*p* < 0.001) whereas baseline HR did not differ between groups (*p* = 0.21). 

#### 3.2.1. Systolic Blood Pressure 

**Figure 1:**
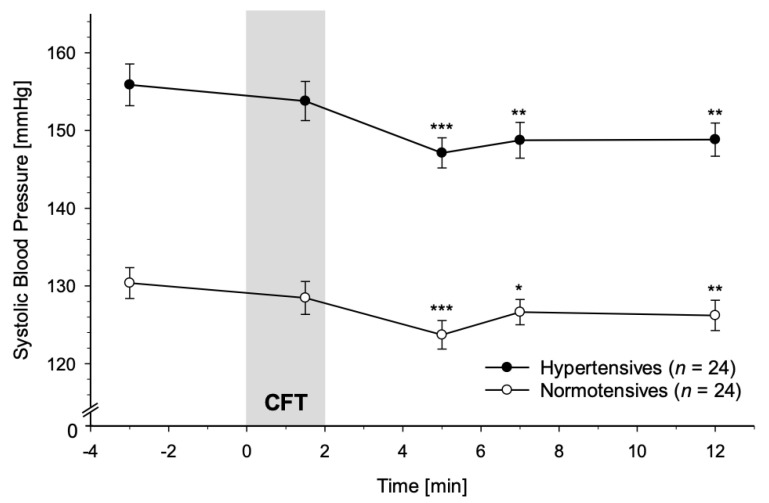
Systolic Blood Pressure (SBP) response to Cold Face Test (CFT; grey bar) in hypertensive participants (black dots) and normotensive controls (white dots) (mean ± SEM). A repeated measures ANOVA revealed that SBP reactivity did not differ between hypertensives and normotensives (interaction group-by-time: *p* = 0.44) except for the higher overall SBP in hypertensives (main effect of group: *p* < 0.001). Repeated measures ANOVAs calculated separately in hypertensives and normotensives revealed main effects of time in both groups (*p*′s < 0.001). Asterisks indicate significant differences between measurement timepoints during/after CFT and the respective baseline levels within each group (* *p* < 0.05; ** *p* < 0.01; *** *p* < 0.001).

HT and NT did not differ significantly in their SBP reactivity to CFT (interaction group-by-time: *p* = 0.44; with age and BMI: *p* = 0.08). However, SBP significantly decreased in response to CFT in both, HT and NT (main effect of time: *F*(3.50, 160.82) = 14.89, *p* < 0.001, partial η^2^ = 0.25, *f* = 0.57), with lowest levels +3 min after CFT-cessation (see Figure 1). This main effect of time was not independent of age and BMI (*p* = 0.11). 

Post-hoc testing revealed (marginally) significant differences from baseline +3 min and +5 min after CFT-cessation across all participants (*p*′s ≤ 0.010). Separate analyses in HT and NT revealed a main effect of time in both groups (HT: *F*(4, 92) = 8.97, *p* < 0.001, partial η^2^ = 0.28, *f* = 0.63; NT: *F*(4, 92) = 6.25, *p* < 0.001, partial η^2^ = 0.21, *f* = 0.52). Moreover, both, HT and NT, displayed significant decreases compared to baseline at all measurement time points after CFT-cessation (*p*′s ≤ 0.013), but not during CFT (*p*′s ≥ 0.30). The expected main effect of group was confirmed (*F*(1, 46) = 80.60, *p* < 0.001, partial η^2^ = 0.64, *f* = 1.32; with age and BMI: *F*(1, 44) = 66.49, *p* < 0.001, partial η^2^ = 0.60, *f* = 1.22; see Figure 1); and post-hoc tests revealed significant group differences for all measurement time points controlling for baseline SBP (*p*′s ≤ 0.046).

Similarly, complementary ANCOVAs using MAP as linear independent variable instead of group, could not detect a significant MAP-by-time interaction (*p* = 0.28; with age and BMI: *p* = 0.07). The expected main effect of MAP was confirmed (*F*(1, 46) = 138.55, *p* < 0.001, partial η^2^ = 0.75, *f* = 1.73; with age and BMI: *F*(1, 44) = 103.08, *p* < 0.001, partial η^2^ = 0.70, *f* = 1.53). However, there was no significant main effect of time (*p* = 0.60; with age and BMI: *p* = 0.92). 

#### 3.2.2. Diastolic Blood Pressure

**Figure 2:**
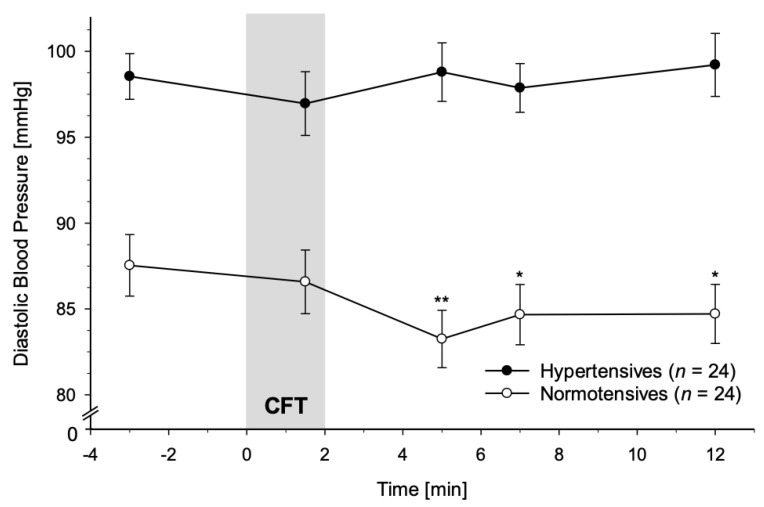
Diastolic Blood Pressure (DBP) response to Cold Face Test (CFT; grey bar) in hypertensive participants (black dots) and normotensive controls (white dots) (mean ± SEM). A repeated measures ANOVA revealed that hypertensives did not show DBP decreases after CFT-cessation as did normotensive controls (interaction group-by-time: *p* < 0.027). Repeated measures ANOVAs calculated separately in hypertensives and normotensives confirmed a significant main effect of time for DBP in normotensives (*p* < 0.002), but not in hypertensives (*p* = 0.59). Asterisks indicate significant differences between measurement timepoints during/after CFT and the respective baseline levels within each group (* *p* < 0.05; ** *p* < 0.01).

As a main finding of our study, HT and NT differed in their DBP reactivity to CFT (interaction group-by-time: *F*(4, 184) = 2.81, *p* = 0.027, partial η^2^ = 0.06, *f* = 0.25; with age and BMI: *F*(4, 176) = 2.03, *p* = 0.092, partial η^2^ = 0.04, *f* = 0.20). As displayed in Figure 2, DBP decreased in NT after CFT-cessation with lowest levels at +3 min after cessation, while in HT, DBP did not decrease. We observed the expected significant group effect for DBP (main effect of group: *F*(1, 46) = 37.99, *p* < 0.001, partial η^2^ = 0.45, *f* = 0.91; with age and BMI: *F*(1, 44) = 38.75, *p* < 0.001, partial η^2^ = 0.47, *f* = 0.94; see Figure 2) whereas the main effect of time was not significant (*p* = 0.23; with age and BMI: *p* = 0.13).

Post hoc tests revealed significant group differences for all measurement time points after CFT-cessation controlling for baseline DBP (+3 to +10 min after CFT-cessation: *p*′s ≤ 0.011). During CFT, HT did not significantly differ from NT in DBP (+1.5 min after CFT-onset: *p* = 0.82), again controlling for baseline DBP. Separate analyses in HT could not detect a main effect of time (*p* = 0.59) whereas separate analyses in NT did (*F*(4, 92) = 4.47, *p* = 0.002, partial η^2^ = 0.16, *f* = 0.44). NT displayed significant decreases compared to baseline for all measurement time points after CFT cession (*p*′s ≤ 0.018), but not during CFT (*p* = 0.30). 

Complementary ANCOVAs using MAP as a linear independent variable instead of group similarly revealed a significant MAP-by-time interaction (*F*(4, 184) = 2.85, *p* = 0.025, partial η^2^ = 0.06, *f* = 0.25; with age and BMI: *F*(4, 176) = 2.14, *p* = 0.078, partial η^2^ = 0.05, *f* = 0.23). Additionally, across all participants, the expected main effect of MAP was confirmed (*F*(1, 46) = 148.38, *p* < *0*.001, partial η^2^ = 0.76, *f* = 1.78; with age and BMI: *F*(1, 44) = 137.31, *p* < 0.001, partial η^2^ = 0.76, *f* = 1.78) and we observed a significant main effect of time (*F*(4, 184) = 3.17, *p* = 0.015, partial η^2^ = 0.06, *f* = 0.25; with age and BMI: *F*(4, 176) = 3.34, *p* = 0.012, partial η^2^ = 0.07, *f* = 0.27). 

#### 3.2.3. Heart Rate

**Figure 3:**
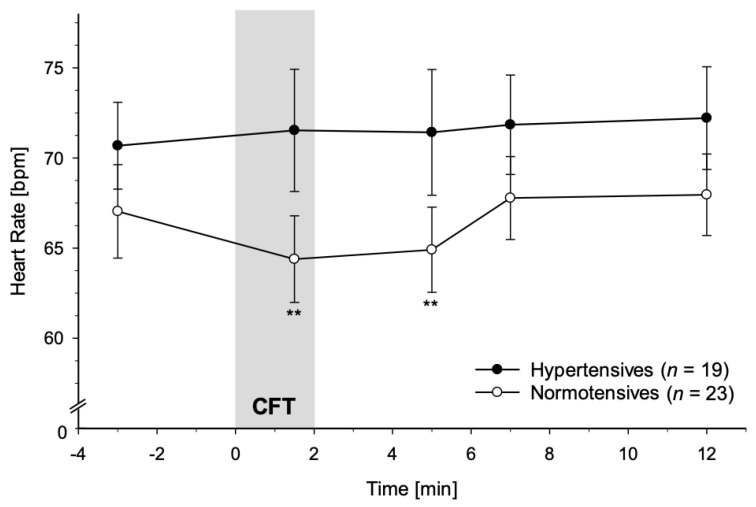
Heart rate (HR) response to Cold Face Test (CFT; grey bar) in hypertensive participants (black dots) and normotensive controls (whites dots) (mean ± SEM). A repeated measures ANOVA revealed that hypertensives failed to show HR decreases in immediate response to CFT as did normotensive controls (interaction group-by-time: *p* < 0.063). Repeated measures ANOVAs calculated separately in hypertensives and normotensives confirmed a significant main effect of time for HR in normotensives (*p* < 0.001), but not in hypertensives (*p* = 0.62). Asterisks indicate significant differences between measurement timepoints during/after CFT and the respective baseline levels within each group (** *p* < 0.01).

HT and NT differed in their HR reactivity to CFT on a marginal significant level (interaction group-by-time: *F*(3.14, 125.49) = 2.46, *p* = 0.063, partial η^2^ = 0.06, *f* = 0.25), but not when controlling for age and BMI (*p* = 0.16). As displayed in Figure 3, HR decreased in NT during CFT but increased after cessation, while in HT, HR did not decrease. 

Indeed, separate analyses in HT and NT confirmed a significant main effect of time in NT (*F*(3.16, 69.51) = 10.26, *p* < 0.001, partial η^2^ = 0.32, *f* = *0*.68), but not in HT (*p* = 0.62). In NT, further post hoc testing revealed that compared to baseline, HR was significantly decreased during CFT and at the first measurement time point after CFT-cessation (+1.5 min after CFT-onset: *p* = 0.005; +3 min after CFT-cessation: *p* = 0.007). Across all participants, HR significantly decreased in response to CFT (main effect of time: *F*(3.14, 125.49) = 5.99, *p* = 0.001, partial η^2^ = 0.13, *f* = 0.39) with marginally significant differences from baseline during CFT (+1.5 min) and +5 min and +10 min after CFT-cessation (*p*′s < 0.09). This main effect of time was not independent of age and BMI (*p* = 0.61). The main effect of group in HR did not reach statistical significance (*p* = 0.15; with age and BMI: *p* = 0.18). 

Complementary ANCOVAs using MAP as linear independent variable instead of group similarly revealed a significant main effect of time without (*F*(3.10, 124.34) = 2.77, *p* = 0.043, partial η^2^ = 0.07, *f* = 0.27) but not with control for age and BMI as confounding variables (*p* = 0.16). We observed a main effect of MAP across all participants (*F*(1, 40) = 4.13, *p* = 0.049, partial η^2^ = 0.09, *f* = 0.31; with age and BMI: *F*(1, 38) = 3.90, *p* = 0.056, partial η^2^ = 0.09, *f* = 0.31). However, there was no MAP-by-time interaction (*p* = 0.12; with age and BMI: *p* = 0.20).

### 3.3. Associations between Chronic Stress and CFT-Induced Reactivity

#### 3.3.1. Systolic and Diastolic Blood Pressure

GLMs with SBP or DBP measures, respectively, as repeated dependent variables, group as categorical variable, and chronic stress as continuous independent variable could not reveal a moderating effect of chronic stress (three-way interactions TICS-CSSS, group, and time for SBP: *p* = 0.80; with age and BMI: *p* = 0.59, for DBP: *p* = 0.64; with age and BMI: *p* = 0.50). There were no significant interactions TICS-CSSS-by-time in terms of SBP (*p* = 0.49; with age and BMI: *p* = 0.72) or DBP (*p* = 0.51; with age and BMI: *p* = 0.71). 

#### 3.3.2. Heart Rate

For HR reactivity, we observed a significant three-way interaction of TICS-CSSS, group, and time (*F*(3.42, 116.32) = 2.66, *p* = 0.045, partial η^2^ = 0.07, *f* = 0.27; with age and BMI: *F*(3.61, 115.60) = 2.24, *p* = 0.075, partial η^2^ = 0.07, *f* = 0.27) when calculating GLMs with HR measures as repeated dependent variable, group as categorical, and chronic stress as continuous independent variable and thus a moderation effect of chronic stress. There was no significant interaction TICS-CSSS-by-time (*p* = 0.27; with age and BMI: *p* = 0.11). 

**Figure 4:**
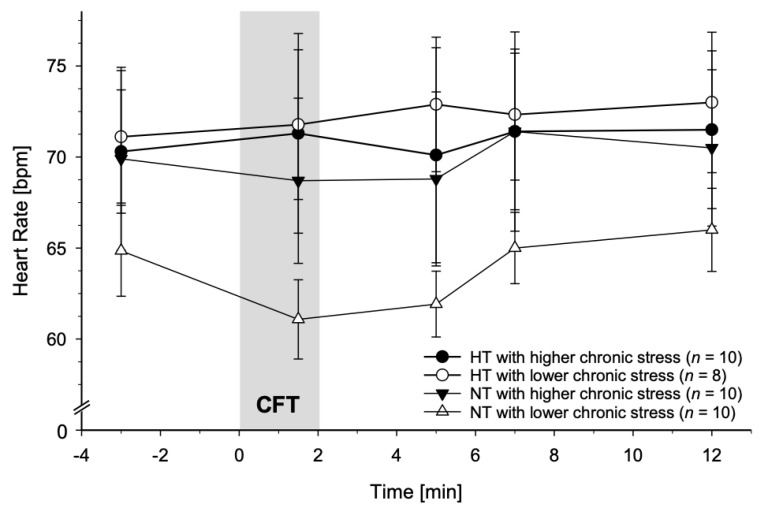
Heart rate (HR) reactivity to Cold Face Test (CFT; grey bar) in hypertensive participants and normotensive controls with higher and lower chronic stress (mean ± SEM). A general linear model revealed that chronic stress moderates HR reactivity to CFT (three-way interaction TICS-CSSS-by-group-by-time: *p* < 0.045) with chronically stressed normotensives resembling the HR reactivity patterns of hypertensives.

HR reactivity in HT and NT with higher and lower chronic stress is illustrated in Figure 4. Whereas HR reactivity profiles of HT seem comparable without marked decreases independent of the amount of chronic stress, NT with higher chronic stress differ from NT with lower chronic stress. More precisely, NT with higher chronic stress displayed HR reactivity profiles without prominent CFT-decreases similar to those of HT. In contrast, NT with lower chronic stress showed a notable decline in HR during CFT which returned to baseline levels after CFT-cessation. 

## 4. Discussion

We (1) investigated cardiovascular reactivity to and recovery from CFT in medication-free hypertensive men as compared to normotensive controls and (2) tested whether chronic stress modulates reactivity to and recovery from CFT. SBP, DBP, and HR were repeatedly assessed before, during, and after CFT. We first found that while NT experienced significant decreases in DBP after CFT-cessation in combination with HR decreases as an immediate response to CFT with subsequent recovery after CFT-cessation, HT did not display significant changes in response to CFT either in terms of DBP or HR. In other words, HT failed to show the “normal” normotensive reactivity or recovery, to CFT in terms of DBP and HR, respectively, showing a rather static picture with reduced up to absent reactivity instead. With respect to SBP, NT and HT exhibited similar reactivity patterns, i.e., decreases in response to CFT, except for the general higher SBP values in HT. Second, depending on hypertension status chronic stress moderated HR reactivity but not SBP or DBP reactivity to CFT. More precisely, regardless of the extent of chronic stress, HT did not exhibit significant HR decreases in response to CFT. While NT with lower chronic stress showed HR decreases with subsequent recovery back to baseline, NT with higher chronic stress resembled the absent reactivity of HT and did not show prominent HR decreases in response to CFT. 

### 4.1. Immediate Reactivity to CFT

The observed expected HR decreases in NT during CFT are in line with previous CFT-studies [14,15,16,17,21,22,38]. Regarding SBP and DBP, NT did not display any changes in their immediate response to CFT in some studies [15,21], while other studies found either increased SBP or DPB [14,16,17]. In all CFT-studies observing immediate increases in SBP and/or DBP in NT, CFT was conducted for a maximum of 60 s with BP decreases starting about 35–45 s after CFT-onset [16,17]. Given that CFT-duration has been found to influence HR decreases [17], extension of CFT-duration as in our study may comparably act on BP resulting in further BP decreases. This might explain why we did not observe BP changes in NT in response to CFT assessed 90 s after onset in our study. Regarding immediate HR reactivity to CFT in HT, the two hitherto published CFT-studies in HT are in line with our findings and observed no immediate HR changes [21,22]. Moreover, in our study, HT did not display any changes in SBP or DBP in immediate response to CFT assessed 90 s after CFT-onset. This result is opposed to the previously reported SBP increases in HT in response to CFT where BP changes were investigated 30–60 s after CFT-onset [21]. We speculate that early BP changes within the first 60 s of the CFT may not persist up to 90 s after CFT-onset. 

### 4.2. Recovery from CFT 

Recovery from CFT has not yet been investigated in humans. Based on the literature presented above, we hypothesized HT to show impaired recovery from CFT. Our results are in line with the hitherto only animal study investigating HR recovery from parasympathetic stimulation in atropine injected and thus PNS inhibited muskrats [39]. 

### 4.3. Moderation by Chronic Stress

Chronically stressed individuals show reduced basal PNS activity [28] and impaired recovery from sympathetic stimulation [27] whereby the PNS is supposed to play a crucial role [40,41]. Similar patterns of autonomic (re)activity are reported in HT [4,5]. So far, associations between chronic stress and reactivity to parasympathetic stimulation have not been investigated. The observed impaired CFT-reactivity in chronically stressed NT and in HT resemble each other and indicate impaired parasympathetic activity with altered reactivity to parasympathetic stimulation in both, chronic stress and hypertension. 

Which mechanisms may underlie our findings? 

#### 4.3.1. SBP and DBP in NT

The observed SBP decreases in NT likely result from acetylcholine (ACh)-induced reduced atrial contractility [42] and diminished ventricle contraction force [43]. We assume the observed DBP decreases in NT to be mediated by the vasodilatory effect of nitric oxide released as a result of M_3_-receptor stimulation by ACh [44] and by the reduced constrictive sympathetic influence on smooth vascular muscles in response to parasympathetic stimulation [45]. Notably, we observed transient BP decreases in our NT immediately after CFT-cessation. Given the CFT-induced sympathetic co-stimulation and resulting peripheral vasoconstriction within the first minute of the CFT [16,17], we speculate that this effect inhibits immediate BP decreases during CFT allowing BP to decrease not before this inhibitory effect disappears. Further, the observed BP increases in our NT during the second half of the recovery period most likely reflect counterregulatory homeostatic feedback mechanisms [46]. 

#### 4.3.2. SBP and DBP Reactivity Divergence in HT vs. NT

The observed similar SBP recovery in NT and HT except for the general higher BP in HT, suggests a functioning SBP recovery in HT. The absence of DBP decreases in HT after CFT-cessation may result from functional impairment of the windkessel function. The windkessel function resembles the ability of the ascending aorta to store part of the blood ejected during systole, forcing it into the peripheral vessels after the aortic valves are closed and thus creating a continuous blood flow [47]. As hypertension is accompanied by endothelial dysfunction, arteriosclerotic stiffening, and calcification of the vessel walls [48], this may impair the relaxation ability of the resistance vessels and thus adjustment ability of the windkessel function in HT [49]. Since the windkessel function accounts for a large extent of the diastolic component of arterial pressure [50], the relaxation capacity of the BP system in the immediate CFT post-exposure phase is likely to be impaired in HT. In line with such reasoning, impairment of endothelium-dependent vasodilatation in essential hypertension has previously been reported [51]. As the elastic capacity of the resistance vessels primarily impacts DBP but not SBP [52], this may also explain why we did not observe the same reactivity patterns for SBP and DBP in HT. 

#### 4.3.3. Decrease in HR in NT and absence of HR Decreases in HT

We propose ACh-induced stimulation of M_2_-receptors in the sinoatrial node to mediate the HR decreasing effect of the CFT [42]. Consequently, the absence of CFT-induced HR decreases in our HT may indicate that parasympathetic reactivity to the CFT is insufficient to elicit HR decreases in HT. Whether this relates to the generally reduced PNS activity in HT remains to be elucidated [3]. Alternatively, dysfunction of M_2_-receptors in HT [53,54] or the persistently heightened sympathetic activity in HT [3] may play a role. With respect to the observed HR increases in NT towards the end of the recovery period, we assume that they result from a compensatory rise of SNS activity after CFT [46]. 

### 4.4. Absence of HR Decreases in Chronically Stressed NT

A likely explanation is reduced vagal inhibitory control in response to parasympathetic stimulation given the decreased basal PNS activity with chronic stress [28]. However, as chronic stress did not relate to BP reactivity to CFT, we assume that effects of chronic stress on endothelial function do not substantially manifest prior to sustained BP elevations [55]. 

With regard to potential *clinical implications,* the observed impaired hemodynamic reactivity to CFT emphasizes the role of the PNS in hypertension in the sense that in reaction to CFT the cardiovascular system shows impaired ability to relax which likely contributes to the sympathovagal imbalance in hypertension. Our findings may point to potential benefits of therapeutic approaches targeting the PNS in hypertensive patients. Whether e.g., direct stimulation of the vagus nerve [56,57] and/or (repeated) application of the non-invasive CFT show beneficial effects in essential hypertensive humans remains to be elucidated. Moreover, the observed absence of HR decreases to CFT in chronically stressed NT may relate to the hypertension risk with chronic stress. 

*Strengths* of our study include the application of the CFT as a standardized short, simple, and non-invasive method to stimulate the PNS, notably without breath holding or facial immersion. Second, the repeated assessment of hemodynamic parameters for up to 10 min after CFT-cessation provides a sufficient time interval for BP and HR kinetic monitoring. Third, we considered effects of chronic stress which may shed light on factors involved in the development of essential hypertension. Finally, we controlled for effects of age and BMI and can thus exclude that the observed reactivity differences of HT and NT result from weight differences and accompanying autonomic changes [36,58]. This is in line with our expectations, as decreased parasympathetic activity has been reported in hypertension even after controlling for BMI [59] and BMI has been shown to be an independent contributor to sympathovagal balance in hypertension development [60]. 

Our study also has its *limitations*. First, generalizability of our results is limited as our study sample comprises normotensive and hypertensive but otherwise healthy and medication-free men. Second, given the effects of age and testosterone levels on vascular functioning in men [61], further investigation in men of different age ranges is warranted. Third, CFT-application stimulates the PNS non-invasively but at the same time co-activates the SNS which does not allow to disentangle pure PNS from mixed PNS plus SNS effects [14,15,17]. Fourth, continuous BP and HR recording would have allowed for a more comprehensive understanding of the reactivity kinetics in response to CFT. Last, we did not assess the full spectrum of assessable SNS and PNS parameters beyond HR and BP such as HRV or pre-ejection period. 

## 5. Conclusions

Essential HT show altered reactivity and recovery patterns in response to CFT in terms of HR and DBP but not in terms of SBP as compared to normotensive controls. These findings indicate impaired ability of the cardiovascular system to relax in essential hypertension. Therefore, therapeutic approaches targeting the PNS might be promising in the treatment of hypertension. Moreover, the observed moderation effect of chronic stress on HR reactivity to CFT may relate to the hypertension risk with chronic stress. Future studies are needed to verify the observed cardiovascular reactivity impairment in essential hypertension and chronic stress and to investigate whether our results extend to further SNS and PNS parameters and other populations. Clinical and therapeutical implications remain to be elucidated.

## Figures and Tables

**Table 1:** Characteristics of study participants.

	Normotensives (*n* = 24) Mean ± SEM (range)	Hypertensives (*n* = 24) Mean ± SEM (range)	*p*
Age (years)	52.71 ± 2.08 (29–64)	54.13 ± 1.25 (38–64)	0.56
BMI (kg/m^2^)	23.82 ± 0.41(20.73–29.04)	26.13 ± 0.51 (21.29–30.76)	0.001 **
MAP (mmHg)	92.80 ± 1.25 (81.67–103.67)	112.94 ± 1.27 (97.83–123.83)	<0.001 ***
Resting SBP ^α^ (mmHg)	122.98 ± 1.46 (105.5–134.0)	149.40 ± 1.64 (133.5–163.5)	<0.001 ***
Resting DBP ^α^ (mmHg)	77.71 ± 1.37 (62.0–89.0)	94.71 ± 1.43 (74.0–107.0)	<0.001 ***
Resting HR ^α^ (min^−1^)	69.52 ± 2.14 (53.0–91.5), *n* = 23	69.96 ± 2.23 (49.0–99.5)	0.89
GSI	0.20 ± 0.04 (0.00–0.89)	0.25 ± 0.04 (0.02–0.72), *n* = 23	0.40
TICS-CSSS	10.19 ± 1.67 (2–34), *n* = 21	11.35 ± 1.50 (0–29), *n* = 23	0.61

BMI = body mass index; DBP = diastolic blood pressure; GSI = Global Severity Index of the Brief Symptom Inventory; HR = heart rate; MAP = mean arterial blood pressure calculated from mean resting BP; *n* = number of participants in case of missing data; *n* = total number of participants; SEM = standard error of the mean; SBP = systolic blood pressure; TICS-CSSS = Chronic Stress Screening Scale of the Trier Inventory for Chronic Stress. α = mean of two resting measurements. *** *p* < 0.001; ** *p* < 0.01.

## Data Availability

The data presented in this study are openly available in PsychArchives at doi:10.23668/psycharchives.4577.
